# Exposure to Unsolvable Anagrams Impairs Performance on the Iowa Gambling Task

**DOI:** 10.3389/fnbeh.2017.00114

**Published:** 2017-06-08

**Authors:** Katrin Starcke, Janet D. Agorku, Matthias Brand

**Affiliations:** ^1^Department of General Psychology, Cognition and Center for Behavioral Addiction Research (CeBAR), University of Duisburg-EssenDuisburg, Germany; ^2^Erwin L. Hahn Institute for Magnetic Resonance ImagingEssen, Germany

**Keywords:** decision making, uncontrollability, stress, learned helplessness, cognition, emotion, motivation

## Abstract

Recent research indicates that external manipulations, such as stress or mood induction, can affect decision-making abilities. In the current study, we investigated whether the exposure to an unsolvable task affected subsequent performance on the Iowa Gambling Task. Participants were randomly assigned to a condition in which they were exposed to unsolvable anagrams (*n* = 20), or a condition in which they worked on solvable anagrams (*n* = 22). Afterwards, all participants played the Iowa Gambling Task, a prominent task that measures decision making under uncertain conditions with no explicit rules for gains and losses. In this task, it is essential to process feedback from previous decisions. The results demonstrated that participants who worked on unsolvable anagrams made more disadvantageous decisions on the Iowa Gambling Task than the other participants. In addition, a significant gender effect was observed: Males who worked on unsolvable anagrams made a more disadvantageous decisions than the other male participants. Females who worked on unsolvable anagrams also made more disadvantageous decision than the other female participants, but differences were small and not significant. We conclude that the exposure to unsolvable anagrams induced the experience of uncontrollability which can elicit stress and learned helplessness. Stress and learned helplessness might have reduced the ability to learn from the given feedback, particularly in male participants. We assume that in real life, uncontrollable challenges that last longer than a single experimental manipulation can affect decision making severely, at least in males.

## Introduction

Weber and Johnson (2009) differentiate decision-making situations according to their degree of uncertainty. They can range from complete ignorance (not even the possible outcomes are known) through uncertainty/ambiguity (the outcomes are known but their probabilities are not) to risk (the outcome probabilities are known), and to certainty (only a single outcome is possible). In neuropsychological decision-making research many studies investigated decision making under ambiguity and risk (Brand et al., 2006). In situations of ambiguity the exact contingencies between options and their outcomes are initially unknown. It is not possible to exactly calculate the advantages and disadvantages of an option on the basis of probabilistic calculations. In these situations, learning from feedback is very important. Examples are the choice of a partner or the choice of holiday locations.

Decisions under initial ambiguity are often simulated with the Iowa Gambling Task (IGT; Bechara et al., 1994, 2000). In this task, participants are exposed to four card decks and can choose one card at a time in 100 trials. Each card selection is associated with a financial gain, but in between, money is lost. Card decks differ in net gains which are unknown to the participants at the beginning of the task and must be learned through the given feedback. However, those decks that initially offer high gains are associated with high losses in the long run and participants must learn that the decks with moderate gains are the most advantageous ones in the long run. It has been proposed that emotional and cognitive processes are both involved in task solution (e.g., Guillaume et al., 2009). Early studies with the IGT investigated decision-making abilities in patients with circumscribed brain lesions, for example in patients with prefrontal cortex or amygdala lesions. Patients with lesions or dysfunctions of the ventromedial prefrontal/orbitofrontal cortex or the amygdala often choose the disadvantageous options in the IGT (Bechara et al., 1999). It was concluded that they lack the ability to experience the rewards and punishments or to integrate previous experiences for upcoming decisions. After these early studies, many patient groups with neurological or psychiatric diseases were examined with the IGT (Dunn et al., 2006; Buelow and Suhr, 2009). For example, patients with basal ganglia dysfunction due to Parkinson’s disease show disadvantageous performance (Kobayakawa et al., 2008, 2010). Deteriorations in patient groups were interpreted as emotional and cognitive deficits to integrate prior consequences into the current decision-making process. In addition, healthy participants were examined with the IGT and their performance has been related with several personality traits and other trait- and state-variables. For example, it has been demonstrated that IGT performance is negatively related to trait anxiety (Miu et al., 2008) and a negative relationship between IGT performance and neuroticism has been found in older adults (Denburg et al., 2009). The IGT is also sensitive to external manipulations such as stress induction (Preston et al., 2007; van den Bos et al., 2009; Wemm and Wulfert, 2017) and mood induction (de Vries et al., 2008). The results indicate that stress overall deteriorates performance in the IGT. Participants in a positive mood perform better in the early trials of the IGT compared to participants in a negative mood.

The current study investigates how the exposure to unsolvable anagrams affects subsequent IGT performance. Unsolvable tasks elicit a psychological state of uncontrollability. Demands that go beyond the capacities of an individual and that are unpredictable or uncontrollable elicit stress (Dickerson and Kemeny, 2004; Koolhaas et al., 2011). Unsolvable tasks have been used as stress induction procedures in recent studies, for example, the Montreal Imaging Stress Task (Dedovic et al., 2005). Participants are exposed to arithmetic tasks that are unsolvable within the given time limit and a fictitious average and expected performance is presented. Participants believe that the tasks should be solvable, but that they are unable to do so. This procedure leads to increases of the stress hormone cortisol. A recent study indicated that those participants who show a high cortisol response also showed an increase in activation in dorsomedial and dorsolateral prefrontal cortex regions (Dedovic et al., 2009b). When their performance was negatively evaluated, their brain activity was reduced in the medial orbitofrontal cortex and the hippocampus. Acute stress induction is proposed to affect decision making in two ways (Starcke and Brand, 2016): increased reward seeking and risk taking due to alterations in dopamine firing rates (motivational and emotional changes); and reduced executive control due to suboptimal prefrontal cortex functioning (cognitive changes). The stress hormone cortisol leads to increased dopaminergic activity (Ungless et al., 2010) which influences reward prediction and feedback learning (Shohamy et al., 2008). Dopaminergic neurons particularly respond towards stimuli that predict high and immediate rewards (Morris et al., 2006; Kobayashi and Schultz, 2008). Focusing on immediate and initially high rewards is dysfunctional in the long run when performing the IGT. Furthermore, stress is supposed to impair executive functions (Hermans et al., 2014) because the release of stress hormones can impair prefrontal cortex functioning. Executive functions are involved in the latter trials of the IGT (Brand et al., 2007).

The exposure to unsolvable tasks not only induces stress, but also learned helplessness (Peterson et al., 1993). Learned helplessness is a psychological state in which individuals experience that none of their actions affects outcomes and they cannot control the situation. Thus, they experience no contingency between action and outcome no matter which action they undertake. Many people react with motivational, emotional and cognitive distortions, i.e., they become passive, depressed, and are unable to discover an adaptive behavioral reaction although an adaptive reaction exists (Seligman, 1975; Maier and Seligman, 1976). According to the theory of learned helplessness, previous experiences of uncontrollability result in the belief that situations are always uncontrollable. As a consequence, individuals who learned to be helpless have difficulties in finding solutions even in situations in which solutions exist. That means, they perform worse than participants who were not exposed to uncontrollable situations before (Hiroto and Seligman, 1975). On a neural level, experimentally induced learned helplessness affects cerebral blood flow in the amygdala and the hippocampus (Schneider et al., 1996). During exposure to unsolvable anagrams, activity in the amygdala increased, while activity in the hippocampus decreased. Altered amygdala activation could affect the experience of rewards and punishments during IGT performance, and decreased hippocampal activation could decrease learning processes in the IGT.

In the current study, we hypothesize that participants who are exposed to unsolvable anagrams subsequently perform worse in the IGT than control participants. The manipulation should induce the experience of uncontrollability which elicits stress and learned helplessness. To the best of our knowledge this is the first study that examined the effect of unsolvable tasks on the IGT in humans. However, recent studies indicate that stress induction with social evaluative stressors affects performance on the IGT (Preston et al., 2007; van den Bos et al., 2009; Wemm and Wulfert, 2017). In addition, a recent animal study reported effects of inescapable footshocks on a rat gambling task (Nobrega et al., 2016). Rats that were exposed to inescapable footshocks showed an increase in disadvantageous choices relative to control rats. Inescapable footshocks reliably induce the experience of uncontrollability in animals with stress and learned helplessness as a consequence.

## Participants and Methods

### Participants

Overall, 54 participants took part in the study. Most of them were students and received course credits for their participation and no financial compensation. The other participants did not receive any course credits and were not paid either. Due to ethical reasons they were asked if they had chronic or acute diseases (including psychiatric diseases), or acute psychological problems. If they affirmed one of these questions they were excluded from participation. The study was approved by the local ethics committee (division of Computer Science and Applied Cognitive Sciences at the Faculty of Engineering, University of Duisburg-Essen) and all participants provided written informed consent. All subjects gave written informed consent in accordance with the Declaration of Helsinki. The protocol was approved by the committee of the division of Computer Science and Applied Cognitive Sciences at the Faculty of Engineering, University of Duisburg-Essen. Half of them were randomly assigned to the experimental group (EG) in which they worked on unsolvable anagrams, and the other half was assigned to the control group (CG) in which they worked on solvable anagrams.

### Methods

#### Solvable and Unsolvable Anagrams

All participants received 20 anagrams with four letters and were required to form a word out of these letters. However, only the anagrams given to the CG were solvable, whereas the anagrams given to the EG were unsolvable. All anagrams were designed in the participants’ native language, i.e., German. Participants were instructed to form one new German word out of each anagram. Given names and homonyms were not allowed. Examples of solvable anagrams were given before the task started. In the CG, solvable anagrams were then presented. An example of a solvable anagram is the word EURE (yours) which can be converted into REUE (regret). In the EG, unsolvable anagrams were presented. An example of an unsolvable anagram is the word KIND (child) which cannot be converted into any other German word. Participants worked 15 min on the anagram task. The anagrams were presented as a paper and pencil task and participants were given all anagrams at once with the time limit of 15 min. They were not allowed to ask any questions within task performance. The anagrams were tested in a pre-study in order to ensure that the solvable anagrams are solvable within the given time limit, but were not so easy that they could be completed immediately. The complete list of anagrams can be seen in Table 1.

**Table 1:** Solvable and unsolvable anagrams.

Solvable anagrams (translation)	Possible solution (translation)	Unsolvable anagrams (translation)
LEIB (body)	Beil (axe)	KIND (child)
BRIE (brie)	Rieb (rubbed)	MORD (murder)
ODEM (breath)	Mode (fashion)	ALSO (thus)
BAUT (builds)	Taub (deaf)	AURA (aura)
RIEF (called)	Reif (ripe)	BANN (ban)
AMTS (official)	Mast (pole)	BAUM (tree)
EGAL (whatever)	Lage (position)	HILF (help)
LIEH (borrowed)	Heil (salvation)	BLUT (blood)
FLAU (slack)	Lauf (run)	HOSE (trousers)
ROTE (red)	Tore (gates)	NOTE (note)
ADLE (ennoble)	Lade (lade)	HAND (hand)
KLEE (clover)	Ekel (disgust)	AUTO (car)
EURE (yours)	Reue (regret)	SINN (meaning)
SIEL (tide gate)	Seil (rope)	BALL (ball)
FEIL (for sale)	Fiel (fell)	WACH (awake)
TORS (genitive of gate)	Rost (rust)	REGE (active)
HELM (helmet)	Mehl (flour)	HUND (dog)
REBE (vine)	Eber (boar)	BAHN (train)
HALM (stalk)	Mahl (meal)	HAUS (house)
EDER (a German river)	Rede (speech)	VERB (verb)

*Translations and solutions are examples of possible translations and solutions*.

#### Decision-Making Performance

All participants performed the computerized version of the IGT (Bechara et al., 2000). In the IGT, participants are exposed to four card decks, A, B, C, and D, and can choose one card at a time (for recent research on construct validity and reliability see Buelow and Suhr, 2009; Buelow and Barnhart, 2017). They are required to gain as much fictitious money as possible and to lose as few money as possible. The task has 100 trials and each card is associated with a financial gain, but in between, money is lost. The contingencies are unknown to the participants, but they receive a feedback after each choice. The exact amount of money that is gained or lost is displayed on the screen (you win/you lose) and visual (smiley or frowny) and acoustic signals (pleasant or unpleasant sound) accompany the feedback. Card decks differ in net gains: Decks A and B are disadvantageous in the long run, whereas decks C and D are advantageous in the long run. Decks A and B offer high gains at the beginning of the task, but during task performance high losses occur in between that exceed the gains in the long run. Decks C and D offer medium gains, but only small losses. This must be discovered by the participants through the processing of the given feedback. To analyze the results, the netscore is calculated: the number of disadvantageous choices is subtracted from the number of advantageous choices. A positive netscore indicates that more advantageous than disadvantageous decisions were made and* vice versa*. The course of the IGT can be subdivided into five blocks of 20 trials. A netscore for each block of trials (1–20, 21–40, 41–60, 61–80, 81–100) can be built.

#### Measurement of Current Affect

To measure current affect prior to and after the experimental manipulation, the Positive and Negative Affect Schedule (Watson et al., 1988) was used. The questionnaire consists of 10 positive and 10 negative adjectives that should be answered on a five point Likert scale from 1 “very few or not at all” to 5 “very much”. Scores were averaged for the positive and the negative affect dimension separately. Thus, results for positive and negative affect can each range from 1 to 5.

#### Measurement of Personality

To measure the big five personality characteristics, the short version of the Big Five Inventory (Rammstedt and John, 2007) was used. The five personality dimensions conscientiousness, neuroticism, extraversion, openness to experiences and agreeableness are assessed with two items each. Items are answered on a five point Likert scale from 1 “not at all” to 5 “completely”. After recoding inverted items scores were averaged for each dimension separately. Thus, scores for each personality dimension can range from 1 to 5. Personality has been assessed to demonstrate that EGs show no major differences in their personality traits.

#### Measurement of General Response Style

To measure the general response style towards dysphoria and depression, the Response Styles Questionnaire (Kühner et al., 2007) was used. The questionnaire consists of 32 items of which 21 measure the response style rumination and 11 measure the response style distraction. Each item can be answered on a four point Likert scale from 1 “nearly never” to 4 “nearly always” and scores are summed up. Scores for rumination can range from 21 to 84, scores for distraction can range from 11 to 44. General response style has been assessed to demonstrate that EGs show no major differences in their general coping styles.

#### Design and Procedure

After providing written informed consent, participants filled out questionnaires on current baseline affect and personality. Then, they were exposed to the experimental manipulation with either solvable or unsolvable anagrams. Affect was measured again after the manipulation. After that, all participants played the IGT. Then, general response style was measured. Finally, demographic variables were assessed and participants were asked if they knew the IGT. All participants were fully debriefed and thanked for participation after they finished the study.

#### Statistical Analyses

Data were analyzed with SPSS version 20 (IBM, Armonk, NY, USA). Differences between the EG and the CG concerning age, response style, and personality were calculated with *t*-tests. Group differences concerning the gender distribution were calculated with a chi-square test (*X*^2^). Potential changes in current affect before and after the experimental manipulation were calculated with repeated-measures analysis of variances (ANOVAs) with “group” as between-factor, and “point in time” as within-factor. The decision-making performance was also calculated with a repeated-measures ANOVA with “group” as between-factor, and “IGT-blocks” as within factor. Greenhouse-Geisser correction was applied when appropriate and partial eta squared ($\eta_{{}_{\text{p}}}^{\text{2}}$) was used as effect size. In order to analyze moderating effects of age, affect and openness to experiences moderated regression analyses were used in which the “IGT netscore” was the dependent variable, and “group” was the predictor. “Age”, “positive and negative affect” after the experimental manipulation, and “openness to experiences” were included as moderating variable in each of the regressions. An ANOVA with “group” and “gender” as factors and “IGT netscore” as dependent variable was performed to analyze potential gender effects.

## Results

### Participants

None of the participants quit the study. However, at the end of the study participants were asked if and if yes from where they knew the IGT. Twelve participants admitted to know the task (mainly from other studies, seven in the EG and five in the CG). They were excluded from further analysis because participants usually receive a debriefing after playing the IGT and thus know the contingencies of the decks. In the EG 20 participants and in the CG 22 participants remained. The excluded participants had a significantly higher IGT netscore than the IGT naive included participants (*p* = 0.005). Of the 42 included participants 30 were females and participants’ age ranged from 18 to 54. Groups did not differ concerning age (mean EG = 21.50, *SD* = 3.25, mean CG = 22.36, *SD* = 7.66, *t* = −0.47, *df* = 40, *p* = 0.64) and gender (EG = 14 females, CG = 16 females, *X*^2^ = 0.04, *df* = 1, *p* = 0.85). Most of the participants studied Applied Cognitive Sciences at the University of Duisburg-Essen (17 in the EG and 16 in the CG) and received course credits for their participation. The other participants were acquaintances of the investigator JDA and studied other fields or worked in a graduate occupation (six), worked in a skilled occupation (two), or went to school (one). Results for response style and personality indicate that groups did not differ, except concerning openness to experiences which is higher in the EG (see Table 2). Results predominantly demonstrate successful randomization.

**Table 2:** Results for personality and response style in both groups.

	EG mean (*SD*)	CG mean (*SD*)	*T*	*df*	*p*
Neuroticism	3.20 (1.01)	2.95 (0.94)	0.82	40	0.42
Extraversion	3.20 (0.83)	3.32 (1.04)	−0.40	40	0.69
Openness	4.40 (0.60)	3.59 (1.10)	3.00	33.06	0.005
Agreeableness	3.05 (0.89)	2.80 (0.98)	0.88	40	0.39
Conscientiousness	3.35 (1.09)	3.18 (0.84)	0.56	40	0.58
Distraction	27.60 (6.33)	25.14 (5.97)	1.30	40	0.20
Rumination	46.80 (11.36)	49.14 (11.00)	−0.68	40	0.50

*EG, experimental group; CG, control group; *SD*, standard deviation*.

### Changes in Current Affect

**Figure 1:**
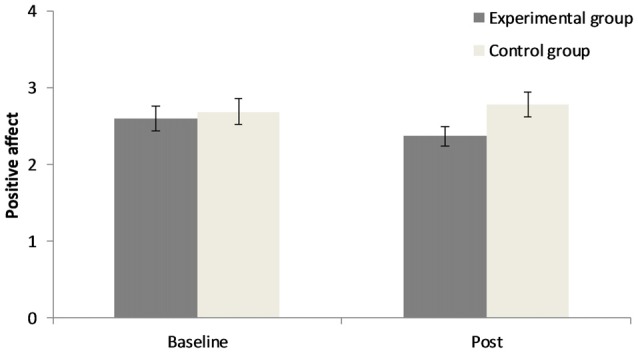
Changes in positive affect before and after the experimental manipulation in both groups. Error bars represent ± one standard error of the mean.

**Figure 2:**
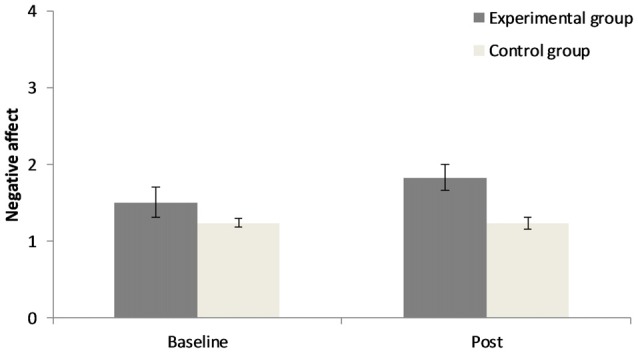
Changes in negative affect before and after the experimental manipulation in both groups. Error bars represent ± one standard error of the mean.

The 2 × 2 repeated measures ANOVA for positive affect demonstrates that there was no significant main effect for “point of measurement”, *F*_(1,40)_ = 0.76, *p* = 0.39, $\eta_{{}_{\text{p}}}^{\text{2}}$ = 0.02, no significant main effect for “group”, *F*_(1,40)_ = 1.44, *p* = 0.24, $\eta_{{}_{\text{p}}}^{\text{2}}$ = 0.04, but a significant interaction of “group” × “point of measurement”, *F*_(1,40)_ = 4.04, *p* = 0.05, $\eta_{{}_{\text{p}}}^{\text{2}}$ = 0.09. Concerning negative affect there was no significant main effect for “point of measurement”, *F*_(1,40)_ = 1.72, *p* = 0.20, $\eta_{{}_{\text{p}}}^{\text{2}}$ = 0.04, a significant main effect for “group”, *F*_(1,40)_ = 8.16, *p* < 0.01, $\eta_{{}_{\text{p}}}^{\text{2}}$ = 0.17, and no significant interaction of “group” × “point of measurement”, *F*_(1,40)_ = 1.82, *p* = 0.19, $\eta_{{}_{\text{p}}}^{\text{2}}$ = 0.04. Results can be seen in Figures 1, 2. In the CG there was a small increase in positive affect, whereas participants in the EG experience a decrease in positive affect after the experimental manipulation. The EG had a higher negative affect than the CG prior to and after the experimental manipulation.

### Decision-Making Performance

**Figure 3:**
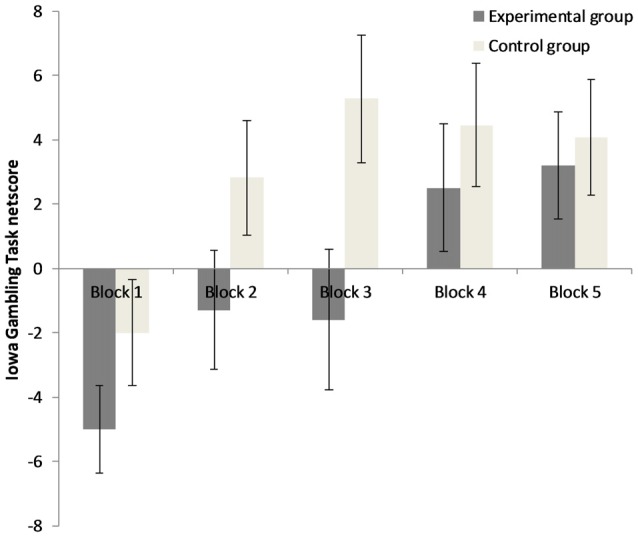
Decision-making performance in both groups for the five blocks of 20 trials. Error bars represent ± one standard error of the mean.

The 5 × 2 repeated measures ANOVA for IGT performance demonstrates that there was a significant main effect for “block”, *F*_(3.13,125.06)_ = 6.98, *p* < 0.001, $\eta_{{}_{\text{p}}}^{\text{2}}$ = 0.15, a significant main effect for “group”, *F*_(1,40)_ = 3.99, *p* = 0.05, $\eta_{{}_{\text{p}}}^{\text{2}}$ = 0.09, but no significant interaction of “group” × “block”, *F*_(3.13,125.06)_ = 1.09, *p* = 0.36, $\eta_{{}_{\text{p}}}^{\text{2}}$ = 0.03. Results can be seen in Figure 3. Results indicate that performance increased during the course of the task in both groups, but that the EG overall performs worse than the CG (mean netscore EG = −2.20, *SD* = 28.76, mean netscore CG = 14.64, *SD* = 25.86). No different learning curves of the EG and the CG were observed as there was no significant interaction between groups and blocks. Age, openness to experiences and post manipulation affect did not moderate the effect of the predictor “group” on “IGT netscore” (*p*s > 0.05). The factor “gender” interacted with the factor “group” (*F*_(1,38)_ = 4.78, *p* < 0.05, $\eta_{{}_{\text{p}}}^{\text{2}}$ = 0.11): Females of the EG and CG differed from one another, but only on a descriptive level (mean netscore EG_females_ = 3.14, *SD* = 31.55, mean netscore CG_females_ = 8.75, *SD* = 23.25, *t*_(28)_ = −0.56, *p* = 0.58). Male participants of the EG performed significantly worse than male participants of the CG (mean netscore EG_males_ = −14.67, *SD* = 16.95, mean netscore_males_ CG = 30.33, *SD* = 27.93, *t*_(10)_ = −3.37, *p* < 0.01). In the CG, there was a trend towards males outperforming females (*t*_(20)_ = −1.84, *p* = 0.08), while in the EG no differences between males and females were observed (*t*_(18)_ = 1.29, *p* = 0.21).

## Discussion

The results of the current study indicate that the exposure to an unsolvable anagram task led to a decrease in decision-making performance in the IGT, which is initially an ambiguous decision-making task. A similar result has been reported for rats in a rat gambling task who were exposed to inescapable footshocks (Nobrega et al., 2016). The exact result pattern of the current study indicates that the individuals exposed to unsolvable anagrams reach a positive IGT netscore during trials 61–80, while control participants already reached a positive netscore during trials 21–40. Brand et al. (2007) postulate, that the early trials of the IGT (1–40) are ambiguous and rely on emotional feedback processing, while the latter trials (41–100) have a more explicit character because contingencies have already been learned by several participants. They demonstrated that only the latter trials are related to executive functions and strategic decision making. However, the shift from ambiguity to explicitness varies from person to person and has no clear cut-point. In the current study, the control participants of our healthy sample showed the typical profile of healthy participants who prefer the advantageous options after a number of trials. In contrast, participants exposed to the unsolvable task show a delayed preference for the advantageous options on a descriptive level. However, the interaction between group and block of trials did not reach significance, so we observed an overall deteriorating effect and no different learning curves. A significant gender effect was observed: Males exposed to the unsolvable anagrams showed worse IGT performance than males in the CG. In females, differences pointed in the same direction, but only on a descriptive level. In the CG, males tended to outperform females. Both findings are in line with recent studies in the field: males are (on a descriptive level) more prone to stress induced deteriorations in the IGT compared with females (Preston et al., 2007) and the relationship between individual stress responses and IGT performance is different for males and females (van den Bos et al., 2009; Wemm and Wulfert, 2017). Under no stress conditions, males make more advantageous decisions than females (van den Bos et al., 2013). We propose that the unsolvable anagrams induced a state of uncontrollability which elicits stress and learned helplessness (Peterson et al., 1993; Dedovic et al., 2005) affecting emotion, cognition and motivation, particularly in our male participants.

Participants who worked on unsolvable anagrams had decreased positive affect after the experimental manipulation and a higher negative affect than control participants from the beginning. This mood state might have interfered with the development of emotional signals that guide the decision process in an advantageous direction. The IGT strongly relies on emotional feedback learning (Bechara et al., 1999). According to the somatic marker hypothesis (Damasio, 1996), somatic signals “mark” the advantageous options even before conscious knowledge about their valence exists. Thus, participants might feel which options are advantageous even before they explicitly know. The reliance on feelings in the IGT appears to be easier when one is currently in a good mood (Suhr and Tsanadis, 2007; de Vries et al., 2008). In the current study, negative affect and reduced positive affect could have reduced emotional feedback learning. However, affect after the experimental manipulation did not moderate the main results.

Participants who were exposed to unsolvable anagrams also might have had reduced cognitive capacities in recognizing response-outcome relations. During the course of the IGT many participants learn which are the advantageous options and know them explicitly at the end of the game (Guillaume et al., 2009). Those participants outperform participants without explicit knowledge. In this case, the task loses its ambiguous character. As mentioned before, during the latter trials of the IGT relationships with other cognitive tasks were observed (Brand et al., 2007). Thus, cognitive abilities play a role in the IGT at the later trials of the task. Classical studies indicate that exposure to uncontrollability leads to disturbances in cognitive tasks (Hiroto and Seligman, 1975; Miller and Seligman, 1975) and a recent study also demonstrated a tendency in that direction (Taylor et al., 2014). Recent research suggests that stress can also lead to reduced performance in cognitive tasks (Starcke et al., 2016). In the current study, cognitive deteriorations might have led to reduced explicit knowledge about the winning and losing probabilities of each deck.

Participants in the unsolvable task condition might also have had motivational deficits. A necessary precondition for successful IGT performance is the motivation to do so. That means, participants must continue their effort in task performance even if they do not recognize the contingencies quickly. Reduced persistence might lead to random deck choices instead of attentive exploration. Motivational deficits are discussed as a feature of learned helplessness (Maier and Seligman, 1976) which means that individuals stop exploring potential solutions too early. Another feature of the IGT is that the options that offer high gains lead to high losses in the long run. Thus, participants have to override the urge to choose options with potential high gains. Under stress, potential high gains have a particularly high salience and potential losses might be ignored (Mather and Lighthall, 2012). The willingness to choose the decks with small, but long-term gains might have been reduced in the EG of the current study.

Participants who worked on unsolvable anagrams might also have been affected by a specific neural pattern. The IGT is thought to rely on numerous brain regions including the ventromedial prefrontal/orbitofrontal cortex, the limbic system (Bechara et al., 1999), and the basal ganglia (Kobayakawa et al., 2008). Learned helplessness and stress have been found to alter activation in these brain regions: Schneider et al. (1996) measured cerebral blood flow with positron emission tomography in participants at baseline and when exposed to solvable and unsolvable anagrams. In the solvable anagram condition, blood flow increased in the hippocampus and decreased in the mammillary bodies, while in the unsolvable condition, blood flow increased in the mammillary bodies and the amygdala and decreased in the hippocampus. A neural pattern like this might alter normal amygdala activation during IGT performance and decrease hippocampal mediated learning abilities. Acute stress also leads to metabolic changes in the prefrontal cortex, limbic system and basal ganglia, and to the secretion of stress hormones such as cortisol (Dedovic et al., 2009a; Pruessner et al., 2010). Excessive cortisol secretion can lead to reduced prefrontal functioning and impair executive functions (Hermans et al., 2014). Increased dopaminergic activity due to cortisol secretion (Ungless et al., 2010) can influence reward prediction and feedback learning (Shohamy et al., 2008). More precisely, stress increases the salience for potential high gains while potential losses are ignored (Mather and Lighthall, 2012).

The above mentioned mechanisms (with the exception of current affect) have not been investigated directly in the current study which limits the conclusions that can be drawn. Future studies should address in more detail the emotional, cognitive, motivational and brain alterations that potentially mediate the effects of unsolvable tasks on decision making. The manipulation check was restricted to changes in current affect. We did not assess the experience of stress and learned helplessness with questionnaires directly because we did not want to evoke the suspicion that the anagrams were unsolvable in the EG. However, more fine grained manipulation checks would be helpful, for example the assessment of physiological and endocrine stress responses, or a questionnaire that measures acute feelings of helplessness in a subtle way. The manipulation with unsolvable anagrams could induce a lot of psychological states such as stress, learned helplessness, ego-depletion, frustration, insecurity, reduced self-esteem, or mood changes. Therefore, it cannot be truly concluded whether the effects reported primarily depend on cognitive, emotional or motivational factors. A further limitation is that the poor IGT performance after the exposure to the unsolvable anagrams can be attributed to the relative small subgroup of male participants. Thus, conclusions can be drawn for males only so far. However, the effects of our female participants point in the same direction, albeit they are smaller and not significant. Future studies should examine equal sized groups of males and females and explore reasons for the gender differences that were observed in the current study. Furthermore, EGs differed concerning their openness to experiences. However, this personality trait did not moderate the main results of the study and recent research also suggests that openness to experiences is unrelated to IGT performance (Denburg et al., 2009).

Current results indicate that the single exposure to an unsolvable task impairs decision-making abilities in an ambiguous situation, particularly in males. In real life, exposure to uncontrollable situations can be long lasting, such as excessive demand in school or job, or continuing failure in finding a job or partner. This might lead to severe deteriorations in real life decision making in ambiguous situations. It is possible that the risk of developing depressive symptoms increases. It has been demonstrated that participants who were exposed to an uncontrollable situation showed cognitive performance similar to patients who suffer from depression (Miller and Seligman, 1975). In extreme cases, people could give up decision making completely. Indecisiveness is included as a diagnostic characteristic of depression in the current Diagnostic and Statistical Manual for Mental Disorders (American-Psychiatric-Association, 2013).

## Author Contributions

KS designed the study and wrote the manuscript; JDA designed the study, collected the data, and analyzed the data; MB designed the study and revised the manuscript.

## Conflict of Interest Statement

The authors declare that the research was conducted in the absence of any commercial or financial relationships that could be construed as a potential conflict of interest.
